# Risk of Atherosclerosis and *Helicobacter pylori* Infection according to CD14 Promotor Polymorphism in Healthy Korean Population

**DOI:** 10.1155/2013/570597

**Published:** 2013-10-21

**Authors:** Sung-Goo Kang, Woo Chul Chung, Sang-Wook Song, Kyu Re Joo, Hyewon Lee, Donghoon Kang, Joune Seup Lee, Kang-Moon Lee

**Affiliations:** ^1^Department of Family Medicine, St. Vincent's Hospital, College of Medicine, The Catholic University of Korea, 93-6 Jungbu-daero, Paldal-gu, Suwon 442-723, Republic of Korea; ^2^Department of Internal Medicine, St. Vincent's Hospital, College of Medicine, The Catholic University of Korea, 93-6 Jungbu-daero, Paldal-gu, Suwon 442-723, Republic of Korea

## Abstract

*Background and Aim*. We aim to elucidate the association of risk factors for atherosclerosis and *H. pylori* infection according to the promotor polymorphism of the CD14 gene in healthy Korean population. *Methods*. The patients who visited our hospital for routine health examinations and 266 healthy adults (170 males and 96 females) were enrolled in this study. The promotor polymorphism at −159C/T of the CD14 gene was determined by PCR-restriction fragment length polymorphism analysis. According to genetic polymorphism and *H. pylori* infection, we analyzed the risk of atherosclerosis. *Results*. The genotype frequencies were CC 7.9%, CT 45.1%, and TT 47.0%, respectively. There were no differences between specific genotypes of CD14 gene and *H. pylori* infection rate. As for HDL cholesterol level, there were significant differences among the three genotypes (*P* < 0.01). In subjects with *H. pylori* infection, no significant differences were observed between specific genotypes of CD14 gene and the risk factors of atherosclerosis. *Conclusion*. The promotor polymorphism at −159C/T of the CD14 gene was associated with the risk factor of atherosclerosis in healthy Korean population. However, it was not associated with the rate of *H. pylori* infection and *H. pylori* induced atherosclerotic risk.

## 1. Introduction 

Clusters of differentiation 14 (CD14) receptor is a mediator of the inflammatory response by recognition of lipopolysaccharide (LPS), a major component of the outer cell wall of Gram-negative bacteria [1]. By inducing inflammatory reactions, chronic infection, such as *Helicobacter pylori* (*H. pylori*), plays an important role in the development of atherosclerosis and its associated complications [2–5]. Recently, it has been widely accepted that inflammation and infection play a key role in atherosclerosis and cardiovascular diseases [6–10]. Clinically, as with *H. pylori* infection, atherosclerotic cardiovascular abnormalities are common, and the infection of this bacterium may be introduced as a risk factor of coronary arterial disease (CAD), independent of known risk factors [2–5]. Moreover, genetic factors for the development of atherosclerosis would be associated with the LPS-mediated activation processes of monocytes and their receptor CD14 [11–13].

The CD14 gene promoter contains a single nucleotide polymorphism, and cytosine (C) to thymine (T) transition polymorphism at position −159 may influence the expression of CD14 [14]. C-to-T transition polymorphism at position −159 in the promoter region of the CD14 gene may affect the affinity of specificity protein (Sp protein) binding and modify transcriptional activity. This variation is important for the pathogenesis of inflammatory disease [14, 15]. It has been thought that a functional polymorphism in the promoter of the CD14 gene (CD14 −159C/T) is associated with *H. pylori*-related gastric carcinoma [16, 17], ischemic heart disease, and atherosclerosis [18–21]. Until now, several studies have yielded conflicting results [22–24]. Because gastric carcinoma and cardiovascular disease are multifactorial disorders caused by the interaction of gene-by-gene and/or gene-by-environment interactions, it is hard to conclude the causal relationship between the specific genotype and the disease. Moreover, in Korean population, there are limited data available on the association of CD14 polymorphism and the specific diseases.

In this study, we focus on normal healthy Korean population and aim to elucidate the association of risk factors for atherosclerosis and* H. pylori* infection according to the promotor polymorphism of the CD14 gene.

## 2. Material and Methods

### 2.1. The Study Population

Healthy patients were enrolled from a teaching hospital of the Catholic University of Medicine, St. Vincent's Hospital, from March 2009 to February 2010. They were asymptomatic examinees of regular health screening with a simple symptom questionnaire at the Health Promotion Center of the same hospital and had endoscopic examinations for free nationwide gastric cancer screening in a Korean adult population. They had no history of hypertension, diabetes, cardiac disease, or any other chronic illness. Individuals with conditions that might have substantial effects on our study results (e.g., serum creatinine >2.5 mg/dL, total bilirubin >3.0 mg/dL), pregnant women, patients with psychiatric diseases, and patients who did not sign a consent form were excluded. Individuals with grossly severe gastric atrophy or ulceration or who were taking specific gastrointestinal medication, NSAIDs, or any other drugs were also excluded. Alcohol drinking was defined as consumption of at least 20 g alcohol/day and up to three times/week. Smoking was defined as current smoker.

### 2.2. Physical Examination and Collection of Specimen

Height, weight, and body mass indices (BMI, kg/m^2^) were measured using the Inbody 720 (Body Composition Analysis, Biospace Co.). The waist circumference was measured with the individual standing upright, with their top raised and under an exhalation state, at the middle area between the lowest area of the number 12 costa and the highest iliac crest, by an experienced person using a ruler with units of 0.1 cm. For systolic and diastolic blood pressure measurements, the subjects took sufficient rest while sitting down over a 5-minute period, and they were measured once with an automatic hemadynamometer.

After fasting for more than 12 hours, serum samples were collected from each patient's upper arm. Fasting blood sugar (FBS), aspartate transaminase (AST), alanine aminotransferase (ALT), total cholesterol, triglyceride, HDL cholesterol, high-sensitivity CRP (hs-CRP), lipoprotein a (Lp (a)), free T4, thyroid stimulating hormone (TSH), and ferritin level were measured. Blood samples (10 mL) were obtained from all subjects and collected in a test tube containing EDTA or heparin.

In enrolled patients, two biopsy specimens were taken during upper gastrointestinal endoscopy from greater curvature side of the midantrum and corpus for histology. The diagnosis of *H. pylori* infection was made by the histologic evidence with a Warthin-Starry silver stain in any of two specimens from antrum and corpus.

## 3. Genetic Polymorphism 

Genomic DNA was acquired from buffy-coat leukocytes using the AccuPrep Genomic DNA Extraction Kit (BIONEER CORPORATION, Daejeon, Republic of Korea). The promoter polymorphism at −159 of CD14 gene was evaluated using the polymerase chain reaction-restriction fragment length polymorphism (PCR-RFLP) method. The PCRs were set up using i-Star Taq DNA polymerase (iNtRON BIOTECHNOLOGY, Seoul, Republic of Korea). In brief, we amplified the CD14 gene promoter region using the forward primer 5′-ATCATCCTTTTCCCACACC-3′ and the reverse primer 5′-AACTCTTCGGCTGCCTCT-3′. In a total reaction volume of 10 *μ*L, 1 *μ*L 10X buffer, 10 pmol of each primer, 1 *μ*L of genomic DNA, and 1 *μ*L of dNTP mixture were combined. The following reaction conditions were established: an initial denaturation at 95°C for 5 minutes followed by 35 cycles at 95°C for 30 seconds, at 64°C for 30 seconds, and at 72°C for 30 seconds. The final extension step was carried out for 5 minutes at 72°C. The reactions were run on a Bio-Rad MyCycler thermal cycler (BIORAD, USA). 3 *μ*L of the resultant PCR products were digested overnight with HaeIII, the appropriate restriction enzyme (New England BioLabs, Beverly, MA, USA), and the digests were electrophoresed in 3% agarose gel. The CD14 C allele was cut into 2 fragments of 140 and 155 base pairs, whereas the T allele remained uncut, with a length of 295 base pairs.

## 4. Statistics

Statistical analysis was conducted using SPSS version 16.0 software. Data was expressed as means ± standard deviation. Allele and genotype frequencies were compared via chi-squared tests. The normality in the distributions was assessed using normal probability plots. ANOVA or Student's *t*-test was used to assess normally distributed variables where appropriate. Kruskal-Wallis H test was used for analysis of factors that were not normally distributed. Differences at the level of *P* < 0.05 were regarded as statistically significant.

## 5. Ethics Statement

Informed consent was obtained from all patients, and the study was approved by the Institutional Review Board of the Catholic University of Korea (VC08TISI0082).

## 6. Results

### 6.1. Basal Characteristics of the Enrolled Patients

A total of 266 healthy adults (170 males and 96 females) were enrolled in this study. The mean age of the subjects was 47.99 ± 10.86 years, and no differences between males and females were detected (male: 47.60 ± 10.49; female: 48.69 ± 11.52; *P* = 0.44). The average BMI of the subjects was 24.33 ± 2.92 kg/m^2^, and the average waist circumference was 88.26 ± 8.24 cm. 

### 6.2. Analysis of Risk Factors for Atherosclerosis according to CD14 Polymorphism

**Figure 1:**
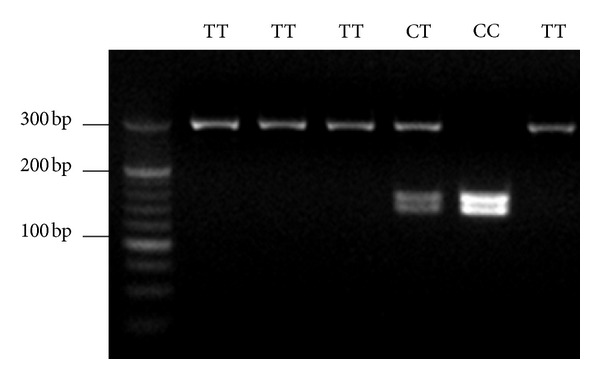
Detection of the −159C/T polymorphism of CD14 gene by using PCR-RFLP. Digestion of the PCR products yielded bands of 295 bp in TT homozygotes, 140 and 155 bp in CC homozygotes, and all 3 bands (140, 155, and 295 bp) in heterozygotes.

Digestion of the PCR products for the promoter polymorphism at −159C/T of the CD14 gene yielded bands of 295 bp in TT homozygotes, 140 and 155 bp in CC homozygotes, and all 3 bands (140, 155, and 295 bp) in heterozygotes (Figure 1). The genotype frequencies were CC 7.9%, CT 45.1%, and TT 47.0%, respectively. There were no differences in age, sex, the ratio of smokers, and alcohol drinkers between the three genotypes.

Between specific genotypes of CD14 gene and the risk factors of atherosclerosis, BMI, waist circumference, systolic blood pressure, diastolic blood pressure, fasting blood sugar, total cholesterol, hs-CRP, and Lp (a), no significant differences were observed. Also, no significant differences of liver function test, white blood cell, thyroid hormone, thyroid-stimulating hormone, and ferritin level existed according to the specific genotypes. However, as for HDL cholesterol level, there were significant differences among the three genotypes (*P* < 0.01 by ANOVA) (Table 1). When post hoc analysis was conducted, CC genotype was clearly associated with higher HDL cholesterol and lower TG level (*P* = 0.04), compared with CT and TT genotypes.

### 6.3. Analysis by Gender

When the subjects were divided by gender, the risk of atherosclerosis according to CD14 polymorphisms was evaluated. In male, there were no differences between specific genotypes of CD14 gene and the risk factors of atherosclerosis (Table 2). In female, no relevant associations were observed except HDL cholesterol and TSH level (*P* = 0.05) (Table 3).

### 6.4. *H. pylori* Infection State and Analysis of Risk Factors for Atherosclerosis in Individuals with *H. pylori* Infection

Total of 133 subjects (50.0%) had associated *H. pylori* infection. There were no significant differences of atherosclerosis risk between *H. pylori*-positive and *H. pylori*-negative subjects.

The frequencies of the CC, CT, and TT genotype with *H. pylori* infection were 10.5% (14/133), 48.9% (65/133), and 40.6% (54/133), and T allele frequency in subjects with *H. pylori* infection was 0.70. No significant difference between specific CD14 genotype and *H. pylori* infection status was found (*P* = 0.06). In subjects with *H. pylori* infection, no significant differences were observed between specific genotypes of CD14 gene and the risk factors of atherosclerosis (Table 4).

## 7. Discussion

Chronic *H. pylori* colonization may be associated with an increased risk for atherosclerosis [25, 26] and plays a causative role in the autoimmune diseases [27]. But the mechanisms by which this bacterium may trigger atherosclerosis remain incompletely understood. The underlying hypothesis is that it has atherogenic capacities by chronic low-grade activation of the hemostasis cascade. CD14 as a pattern recognition molecule of the innate immune response is the main receptor for LPS generated by Gram-negative bacteria such as *H. pylori*. It stimulates the release of proinflammatory cytokines, which is involved in the induction of the synthesis of tumor necrosis factor-*α* (TNF-*α*), interleukin-1 (IL-1), interleukin-6 (IL-6), growth factors, coagulation factors, and primary immune response. Activated macrophages via CD14 receptor may induce IL-6 production within the atheroma. An abnormally high plasma level of IL-6 represents a further risk factor for plaque rupture and atherosclerosis progression [10, 11, 28, 29].

Previously, it has been reported that acute myocardial infarction/atherosclerosis are more closely related to CD14 −159TT homozygotes [30–32]. Meta-analysis reported that the causal relationship between TT genotype and ischemic heart disease was probably established only in the East Asian population, but there was no significant association in a European population and an Indian population [33]. In our study, 47.0% of the participants evidenced the TT genotype, which is substantially higher than the 15.6–27.4 % reported in studies conducted in other countries [14–16, 18, 19, 22]. It suggested that the contribution of genetic determinants might differ significantly between ethnicities. For the most part, the ischemic heart disease has complex origins that are caused by a combination of genetic, environmental, and lifestyle factors. Therefore, it is hard to conclude that the single gene variants will be proven as probable association with the specific disease. In this study, we focused on the relation between a risk factor and genetic polymorphism, and selected healthy adults without specific gastrointestinal symptoms to reduce the risk of selection bias. Our data showed that −159CT and TT genotypes of CD14 gene have lower HDL cholesterol and higher triglyceride serum concentrations as compared to CC genotype. As demonstrated previously, low HDL cholesterol was well known as a potent risk factor even in the presence of very low level of LDL cholesterol [34]. Evidence of the association between triglyceride values and cardiovascular diseases is continuously being accumulated [35–37].

Although this study includes small size of the study population, it might be difficult to figure out the association between genetic polymorphism and disease phenotype. Also, we did not consider risk factors such as exercise activity and diet pattern. Especially, these are the important modifiable and preventable risk factors for cardiovascular disease. Our result showed that significant difference of HDL cholesterol level was pronounced in female population, because of the possibility that these contributing factors for atherosclerosis were excluded in female. In our results, there was significant difference in TSH level among the three genotypes. Although the high level of serum TSH level is associated with multivessel disease, it was not the determinant of cardiovascular disease in patients with normal range of thyroid function [38, 39]. Moreover, it cannot exclude a type 2 statistical error. To overcome it, the researcher will sample as many subjects as cost and time allow.

In the last two decades, epidemiologic studies have demonstrated that atherosclerosis is associated with several infectious pathogens, including *H. pylori*, and the existence of a positive association between *H. pylori* and CAD [2–5]. In addition, a modest influence on CAD and progressive atheroma could be caused by *H. pylori* infection [40, 41]. However, it could be debatable due to confounding bias and influenced by the degree of investigations heterogeneity. On the other hand, individual susceptibility for specific disease existed, and genetic polymorphism can be associated with pathogenetic mechanism. Previously, specific genotype of CD14 gene may favor increased inflammation in atheroma, promoting possible worsening atherosclerosis [18–22]. We hypothesized that genetic susceptibility to *H. pylori* infection is associated with the risk of atherosclerosis and aimed to evaluate risk factors for atherosclerosis according to the CD14 polymorphism in healthy subjects with *H. pylori* infection. In our results, the specific genotype of CD14 gene seems to have the risk of atherosclerosis. However, CD14 polymorphism was associated neither with the rate of* H. pylori* infection nor with *H. pylori* induced atherosclerotic risk response in Korean population. There were several limitations in this study. We could not rule out the possibility that some cases were negative for *H. pylori* at the time of collection of tissue sample. Also, we did not consider the specific pathogenic strain because the association of chronic *H. pylori* infection with risk of atherosclerosis appeared to be limited to cagA bearing strains in previous study [42, 43]. Its clinical significance is uncertain and these results should expand the sample to do further research. In addition, whether eradication of *H. pylori *might decrease the risk of atherosclerosis should be evaluated.

To date, there are limited data regarding the probability of cardiovascular diseases according to CD14 genetic polymorphisms in Korean population. This provides a clue that the high risk individuals of cardiovascular diseases are discriminated using genetic analysis. In conclusion, the promoter polymorphism at −159C/T of the CD14 gene is positively associated with the risk factor of atherosclerosis in healthy Korean population. Broader studies will be required before any relatively concrete conclusions can be drawn.

## Figures and Tables

**Table 1:** Risk factors of atherosclerosis according to CD 14 −159 genotypes.

	CC	CT	TT	P*
Age	47.00 ± 11.95	47.56 ± 11.43	48.44 ± 10.04	0.75
Sex (male : female)	11 : 10	80 : 40	79 : 46	0.44
Smoking	12	50	42	0.09
Alcohol drinking	14	72	68	0.47
*H. pylori* infection	14	65	54	0.06
Waist circumference (cm)	87.52 ± 7.97	88.88 ± 8.65	87.79 ± 7.93	0.58
Body mass index (Kg/m^2^)	24.11 ± 2.83	24.34 ± 2.99	24.37 ± 2.91	0.94
SBP (mmHg)	125.28 ± 18.59	126.63 ± 13.52	127.07 ± 14.17	0.88
DBP (mmHg)	75.44 ± 12.76	75.32 ± 9.10	76.23 ± 9.87	0.78
Hemoglobin (g/dL)	14.37 ± 1.35	14.68 ± 1.88	14.59 ± 1.60	0.72
ESR	15.27 ± 1.15	12.84 ± 1.04	13.70 ± 1.03	0.76
AST (IU/L)	24.90 ± 9.12	24.54 ± 9.74	23.05 ± 7.06	0.33
ALT (IU/L)	24.07 ± 15.27	28.36 ± 19.11	26.14 ± 14.12	0.41
rGTP (IU/L)	30.81 ± 31.82	42.44 ± 47.29	36.10 ± 33.00	0.31
FBS (mg/dL)	96.24 ± 11.49	102.22 ± 21.71	103.01 ± 25.21	0.45
Uric acid (mg/dL)	5.43 ± 1.34	5.57 ± 1.54	5.33 ± 1.45	0.48
Triglycerides (mg/dL)	105.38 ± 48.85^†^	159.04 ± 123.83	160.76 ± 97.53	0.08
Total cholesterol (mg/dL)	191.52 ± 31.01	204.02 ± 39.22	194.77 ± 32.89	0.13
HDL cholesterol (mg/dL)	52.67 ± 15.04^†^	45.87 ± 9.61	44.10 ± 9.19	<0.01*
hs-CRP (mg/dL)	0.23 ± 0.23	0.19 ± 0.40	0.21 ± 0.40	0.92
Lp (a)	11.93 ± 9.92	11.74 ± 13.09	15.92 ± 17.52	0.35
Free T4 (ng/dL)	0.98 ± 0.17	1.03 ± 0.15	1.16 ± 0.91	0.23
TSH (*μ*g/mL)	2.14 ± 1.79	1.89 ± 1.97	1.70 ± 2.68	0.20
Ferritin (ng/mL)	133.00 ± 164.03	134.39 ± 88.13	145.43 ± 129.98	0.87

All values are presented as mean ± standard deviation of the mean. *P values were obtained by one-way ANOVA. ^†^ *P* values (CC genotype versus CT/TT genotypes) were obtained by Scheffe's post hoc test after one-way ANOVA (*P* < 0.05).

*H. pylori*:* Helicobacter pylori*, SBP: systolic blood pressure, DBP: diastolic blood pressure, ESR: erythrocyte sedimentation rate, AST: aspartate transaminase, ALT: alanine aminotransferase, FBS: fasting blood sugar, hs-CRP: high-sensitivity C-reactive protein, Lp (a): lipoprotein a, and TSH: thyroid stimulating hormone.

**Table 2:** Risk factors for atherosclerosis according to male gender.

	CC	CT	TT	P*
Age	44.18 ± 7.25	47.14 ± 11.35	48.77 ± 9.97	0.29
Waist circumference (cm)	86.68 ± 8.73	89.63 ± 8.41	88.75 ± 7.20	0.64
Body mass index (Kg/m^2^)	25.28 ± 2.82	24.73 ± 2.94	24.85 ± 2.62	0.82
SBP (mmHg)	131.00 ± 15.61	128.19 ± 12.39	129.19 ± 13.09	0.67
DBP (mmHg)	79.20 ± 13.27	76.18 ± 9.13	78.07 ± 9.35	0.46
Hemoglobin (g/dL)	15.50 ± 0.62	15.34 ± 1.90	15.36 ± 1.06	0.46
ESR	13.00 ± 5.29	11.28 ± 7.88	11.00 ± 6.60	0.60
AST (IU/L)	27.91 ± 9.83	25.31 ± 8.67	24.65 ± 7.43	0.70
ALT (IU/L)	29.27 ± 18.93	31.70 ± 18.87	30.30 ± 14.93	0.68
rGTP (IU/L)	41.64 ± 39.71	53.42 ± 53.89	46.39 ± 36.35	0.38
FBS (mg/dL)	99.09 ± 12.68	104.88 ± 24.60	105.43 ± 25.93	0.82
Uric acid (mg/dL)	6.11 ± 1.34	6.03 ± 1.49	5.95 ± 1.32	0.95
Triglycerides (mg/dL)	109.09 ± 50.32	179.51 ± 141.44	171.10 ± 110.21	0.09
Total cholesterol (mg/dL)	189.73 ± 31.04	205.79 ± 39.91	200.51 ± 29.52	0.51
HDL cholesterol (mg/dL)	49.27 ± 15.32	44.30 ± 9.45	42.68 ± 9.54	0.51
hs-CRP (mg/dL)	0.30 ± 0.28	0.24 ± 0.40	0.20 ± 0.40	0.30
Lp (a)	7.26 ± 9.95	9.41 ± 9.60	14.36 ± 20.07	0.41
Free T4 (ng/dL)	1.00 ± 0.16	1.04 ± 0.15	1.21 ± 1.12	0.45
TSH (*μ*g/mL)	3.06 ± 3.31	1.60 ± 1.24	1.85 ± 3.27	0.50
Ferritin (ng/mL)	190.86 ± 208.36	164.29 ± 82.94	183.24 ± 145.98	0.84

All values are presented as mean ± standard deviation of the mean. *P values were obtained by Kruskal-Wallis *H* test.

SBP: systolic blood pressure, DBP: diastolic blood pressure, ESR: erythrocyte sedimentation rate, AST: aspartate transaminase, ALT: alanine aminotransferase, FBS: fasting blood sugar, hs-CRP: high-sensitivity C-reactive protein, Lp (a): lipoprotein a, and TSH: thyroid stimulating hormone.

**Table 3:** Risk factors for atherosclerosis according to female gender.

	CC	CT	TT	P*
Age	50.10 ± 15.44	48.40 ± 11.69	47.84 ± 10.25	0.93
Waist circumference (cm)	85.09 ± 6.74	87.16 ± 9.07	86.00 ± 8.96	0.79
Body mass index (Kg/m^2^)	22.64 ± 2.20	23.45 ± 2.94	23.49 ± 3.24	0.83
SBP (mmHg)	118.12 ± 20.49	123.09 ± 15.39	123.05 ± 15.43	0.65
DBP (mmHg)	70.75 ± 11.11	73.35 ± 8.85	72.74 ± 10.00	0.77
Hemoglobin (g/dL)	13.13 ± 0.61	13.33 ± 0.83	13.26 ± 1.49	0.82
ESR	18.00 ± 16.63	16.53 ± 14.37	17.89 ± 13.38	0.96
AST (IU/L)	21.60 ± 7.44	22.95 ± 11.58	20.30 ± 5.42	0.65
ALT (IU/L)	18.30 ± 7.04	21.51 ± 17.93	18.98 ± 8.98	0.96
rGTP (IU/L)	18.90 ± 14.05	19.90 ± 11.49	18.41 ± 14.30	0.59
FBS (mg/dL)	93.10 ± 9.69	96.77 ± 12.65	98.96 ± 23.63	0.85
Uric acid (mg/dL)	4.58 ± 0.73	4.53 ± 1.11	4.17 ± 0.86	0.29
Triglycerides (mg/dL)	111.30 ± 64.14	110.87 ± 54.75	114.57 ± 62.84	0.97
Total cholesterol (mg/dL)	193.50 ± 32.53	200.38 ± 38.01	184.91 ± 36.23	0.18
HDL cholesterol (mg/dL)	57.40 ± 14.51	49.36 ± 9.46	46.11 ± 7.86	0.05*
hs-CRP (mg/dL)	0.15 ± 0.13	0.08 ± 0.05	0.24 ± 0.58	0.35
Lp (a)	18.95 ± 4.56	17.26 ± 18.15	18.33 ± 12.75	0.46
Free T4 (ng/dL)	0.96 ± 0.19	1.00 ± 0.16	1.07 ± 0.17	0.10
TSH (*μ*g/mL)	1.49 ± 2.06	2.56 ± 2.96	1.43 ± 2.37	0.05*
Ferritin (ng/mL)	63.58 ± 44.21	58.77 ± 45.86	80.91 ± 56.66	0.42

All values are presented as mean ± standard deviation of the mean. *P values were obtained by Kruskal-Wallis *H* test.

SBP: systolic blood pressure, DBP: diastolic blood pressure, ESR: erythrocyte sedimentation rate, AST: aspartate transaminase, ALT: alanine aminotransferase, FBS: fasting blood sugar, hs-CRP: high-sensitivity C-reactive protein, Lp (a): lipoprotein a, and TSH: thyroid stimulating hormone.

**Table 4:** Risk factors for atherosclerosis according to CD 14 −159 genotypes in subjects with *H. pylori* infection.

	CC	CT	TT	P*
Age	43.15 ± 7.16	47.31 ± 10.45	46.24 ± 9.80	0.38
Waist circumference (cm)	89.36 ± 7.67	88.98 ± 8.91	87.05 ± 7.29	0.42
Body mass index (Kg/m^2^)	24.85 ± 2.66	24.21 ± 3.09	24.14 ± 2.93	0.73
SBP (mmHg)	127.85 ± 15.15	125.35 ± 13.58	125.31 ± 12.96	0.82
DBP (mmHg)	78.00 ± 11.80	75.27 ± 8.82	75.67 ± 9.52	0.64
Hemoglobin (g/dL)	14.90 ± 1.23	14.91 ± 1.45	14.54 ± 1.65	0.39
ESR	17.71 ± 1.33	14.70 ± 1.44	16.74 ± 1.15	0.83
AST (IU/L)	28.38 ± 9.80	24.19 ± 10.15	23.78 ± 5.68	0.33
ALT (IU/L)	28.23 ± 18.10	26.50 ± 16.39	28.30 ± 13.54	0.21
rGTP (IU/L)	38.85 ± 37.65	49.73 ± 59.32	32.28 ± 24.49	0.31
FBS (mg/dL)	99.23 ± 11.53	103.67 ± 24.49	101.74 ± 22.84	0.78
Uric acid (mg/dL)	5.85 ± 1.30	5.44 ± 1.51	5.47 ± 1.66	0.68
Triglycerides (mg/dL)	105.92 ± 49.02	164.89 ± 108.10	151.50 ± 108.60	0.18
Total cholesterol (mg/dL)	199.92 ± 29.42	201.55 ± 38.49	196.39 ± 29.44	0.72
HDL cholesterol (mg/dL)	50.15 ± 13.47	45.16 ± 8.62	44.98 ± 9.91	0.20
hs-CRP (mg/dL)	0.28 ± 0.25	0.16 ± 0.18	0.27 ± 0.56	0.59
Lp (a)	9.82 ± 11.34	13.19 ± 16.07	12.26 ± 14.86	0.88
Free T4 (ng/dL)	0.96 ± 0.16	1.01 ± 0.14	1.08 ± 0.32	0.16
TSH (*μ*g/mL)	3.34 ± 3.31	1.59 ± 1.32	2.00 ± 3.88	0.13
Ferritin (ng/mL)	168.80 ± 181.46	152.82 ± 96.51	110.18 ± 53.60	0.26

All values are presented as mean ± standard deviation of the mean. *P values were obtained by one-way ANOVA.

SBP: systolic blood pressure, DBP: diastolic blood pressure, ESR: erythrocyte sedimentation rate, AST: aspartate transaminase, ALT: alanine aminotransferase, FBS: fasting blood sugar, hs-CRP: high-sensitivity C-reactive protein, Lp (a): lipoprotein a, and TSH: thyroid stimulating hormone.
